# Endothelin-1 Levels in Scleroderma Patients: A Pilot Study

**DOI:** 10.1155/2013/125632

**Published:** 2013-07-30

**Authors:** Emanuele Cozzani, Sanja Javor, Erika Laborai, Massimo Drosera, Aurora Parodi

**Affiliations:** DISSAL, Section of Dermatology, IRCCS University Hospital San Martino-IST, 16132 Genoa, Italy

## Abstract

Endothelin-1 (ET-1) is a potent endogenous vasoconstrictor, which mediates vascular wall cells proliferation, fibrosis, and inflammation through two types of ET-1 receptors (ET-A and ET-B). In our retrospective study the serum levels of ET-1 in 18 systemic sclerosis (SSc) patients with and without digital ulcers (DUs) were assessed to observe possible correlation between the levels of ET-1, the evolution of SSc, and the therapy with an ET-1 antagonist (bosentan). In all our patients, the levels of ET-1 were found higher than normal range and correlate with the severity of the disease. Furthermore we also observed that in patients without DUs the levels of ET-1 were higher and did not correlate with new DUs development. In conclusion, the levels of ET-1 in our studied patients do not correlate with the possible development of DUs. The reduction of ET-1 levels in DUs patients in therapy with bosentan confirms the efficacy of this molecule both for treatment and prevention of digital ulcers. The inhibition of ET-A receptor by its antagonist may activate the opposite ET-B receptors, with well-known function ET-1 degradation and reducing of ET-1 serum level as confirmed in our pilot study.

## 1. Introduction

Systemic sclerosis (SSc) is an autoimmune connective tissue disease characterized by vascular involvement and fibrosis of different tissues and organs. Approximately 50% of patients with SSc develop digital ulcers (DUs) during the course of the disease [1]. An important role in the pathogenesis of SSc is played by the endothelins and in particular by endothelin 1 (ET-1). ET-1 is a potent endogenous vasoconstrictor, which also mediates vascular wall cells proliferation, fibrosis, and inflammation, mainly synthesized in endothelial cells with autocrine and paracrine effects. There are two types of ET-1 receptors: ET-A and ET-B. ET-A receptors are expressed by vascular smooth muscle cells and mediate vasoconstriction. ET-B receptors are mainly expressed on endothelial cells and mediate vasodilatation. In SSc patients, ET-B receptors are downregulated. This fact may displace the balance towards vasoconstriction and fibrosis [2]. The aim of our retrospective study was to assess the serum levels of ET-1 in SSc patients with and without digital ulcers, in order to observe a possible correlation between the levels of ET-1 and the evolution of SSc. In addition, we evaluated the efficacy of the bosentan, a dual ET-1 receptor antagonist licensed for the management of pulmonary arterial hypertension (PAH) and the prevention of DUs [3].

## 2. Methods

### 2.1. Subjects

Retrospectively, we examined 18 female patients with systemic sclerosis (mean age 68 years), attending the Department of Dermatology at the University of Genoa. The clinical diagnosis was confirmed by serological data (antinuclear antibodies and antiextractable nuclear antigens antibodies). Classification at the diagnosis for each patient was made according to the guidelines of the American College of Rheumatology [4]: 14 patients (4 with PAH) had limited cutaneous SSc (LcSSc), 3 patients (1with PAH) had diffuse cutaneous SSc (DfSSc) and 1 patient had calcinosis, Raynaud phenomenon, esophageal dysmotility, sclerodactily and teleangectasia (CREST syndrome).

The dosage of the ET-1 was evaluated retrospectively on the first serum (collected at the diagnosis) and the last serum available (average of 6 years after) to evaluate if the dosage of ET-1 collected at the first visit in our department was able to herald possible complications during the years (PAH, DUs, pulmonary fibrosis, etc.). In 5 patients bosentan was prescribed for PAH assessed by transthoracic echocardiography B-mode. In these 5 patients ET-1 was studied before bosentan treatment and after 3 months or more. During six years of follow-up 7 out of 18 patients developed at least one digital ulcer; five patients were treated with calcium antagonist (CA) and two with bosentan. All the 7 patients with digital ulcers were assessed by dermatological evaluation monthly and capillaroscopy every six months.

### 2.2. Serum Specimens

Blood was collected from 18 patients in tubes and centrifuged obtaining sera specimens. The sera had been frozen at −20°C. In 13 we took the first serum at the time of the diagnosis of SSc and the second serum available 6 years after. For 5 patients treated with bosentan ET-1 sera were collected before and 3 months or more after the specific treatment. Finally, we collected a total of 36 sera.

### 2.3. Determination of ANA and Anticentromere Antibodies

All sera were tested by IIF by using HEp-2000 cells as substrate (Immuno Concepts, Sacramento, CA, USA, distributed by Technogenetics Bouty Group, Milan, Italy) at the basic dilution of 1/40. When positive, the sera were diluted in order to obtain the final titers.

### 2.4. Qualitative Determination of Anti-ENA

All sera were screened by ENAscreen (ORGENTEC Diagnostika GmbH, Germany), an ELISA test system for the qualitative screening of immunoglobulin G (IgG) class autoantibody against extractable nuclear antigens (ENAs) in human serum or plasma. A mixture of purified antigens SS-A, SS-B, Sm, RNP/Sm, Scl 70, and Jo-1 was coated onto microwells. Antibodies against the coated antigen, when present in diluted 1 : 100 patient sample, bind to the respective antigen.

### 2.5. Semiquantitative Determination of Anti-ENA

All sera were screened by ENAcombi (ORGENTEC Diagnostika GmbH, Germany), an immunometric test for the semiquantitative screening of anti-ENA IgG. Microwells were coated with purified antigens of SS-A, SS-B, Sm, RNP/Sm, Scl 70 and Jo-1. Antibodies against the coated antigen, when present in diluted 1 : 100 patient sample, they bind to the respective antigen. Cut-off value was 25 (U/mL).

### 2.6. Determination of Endothelin-1

ET-1 sera titers were evaluated by enzyme-linked immunometric assay (ELISA; EIA Enzo Life Sciences) which uses well coated with a monoclonal antibody specific for ET-1. ET-1 titer was considered normal in the range 0,1–3 pg/mL.

## 3. Results

In all our patients the levels of ET-1 were found higher than normal range (0,1–3 pg/mL), with values between 3,2 and 24 pg/mL (Table 1). The highest ET-1 level (24 pg/mL) was found in the first serum of the patient with SSc with greater skin and systemic involvement. On the contrary, the level of ET-1 close to the normal level (3,2 pg/mL) was found in a patient with a slight disease involvement. Ten patients were positive for anticentromere antibodies (Table 1). Anti-ENA antibodies were found in 7 out of 18 studied patients who showed ET-1 concentrations slightly higher compared with anti-ENA antibodies negative patients. As for the patients treated with bosentan, all of them presented a reduction of ET-1 levels after the treatment period. The major benefit was found in patients treated for nine months. In particular one patient presented a reduction of ET-1 levels (from 8,1 to 6,9 pg/mL) that were associated with the improvement of clinical features and with the resolution of digital ulcers. By contrast, we observed that a short therapy with bosentan (3 months) induces minimal decrease of ET-1 levels without any apparent clinical changes (patient number 7). All the patients with DUs showed an “advanced” capillaroscopic pattern characterized by severe loss of capillaries with extensive avascular areas, irregular enlargement of the capillaries, and neoangiogenesis at the last stage of the disease. In LcSSc patients the ulcers appear about 10 years after the diagnosis of the disease and in DfSSc patients after about 5 years. Patients with DUs treated with bosentan maintained microvascular alterations without progression, followed by significant improvement of cutaneous lesions reflecting a therapeutic role of bosentan. During the treatment with bosentan no patient developed new DUs. Moreover, patients numbers 1 and 5 treated with bosentan showed a complete clinical improvement of digital ulcers and remission of pain in about 3 months correlated with a reduction of ET-1 serum levels (15% and 23%, resp.) and none developed new DUs in accordance with data of the literature [5]. On the contrary DUs patients treated with calcium antagonist (patients: numbers 3, 8, 10, 12, and 13) had a slightly longer clinical remission. In addition the 5 PAH patients (patients: numbers 1, 4, 5, 7, and 9) showed a stabilization of pulmonary pressure.

## 4. Discussion

According to recent reports [6] our pilot study confirmed that scleroderma patients present with ET-1 sera levels higher than normal range and these concentrations seem to correlate with the severity of the disease. Does it mean that high ET-1 levels at the onset of the disease would predict a more severe course of SSc? In all 18 patients no correlation between age, ANA titers, anti-centromere titers, and ET-1 levels was found. Patients with ET-1 >5 pg/mL at the first sera had also anti-SS-A antibodies positivity, except patient number 1 who presented with ET-1 at 8,1 pg/mL and only anti-Scl 70 antibodies positivity. Patients with anti-SS-A antibodies showed a more severe subset, a faster progression of the sclerosis, and a constant involvement of lungs [7]. So, in the patients with ET-1 >4 pg/mL and SS-A positivity, the clinical course of the disease was worse. Furthermore we also observed that in patients without DUs the level of ET-1 was higher and did not correlate with DUs development. In conclusion the levels of ET-1 in our patients do not correlate with the possible development of DUs. The reduction of ET-1 levels in DUs patients in therapy with bosentan confirms the efficacy of this molecule both for treatment and prevention of digital ulcers. Previous studies reported that oral bosentan 125 mg twice a day significantly reduces the occurrence of new digital ulcers and was well tolerated [8] with no refractory ulcers while taking bosentan [9]. The inhibition of ET-A receptor by its antagonist may activate the opposite ET-B receptors, with well known function of ET-1 degradation and reducing ET-1 serum level as confirmed in our pilot study. However, these data need to be confirmed with future studies involving a larger number of patients with systemic scleroderma.

## Figures and Tables

**Table 1:** Correlation between clinical subsets (DfSSC, LcSSc, CREST, and overlap syndrome), presence of ulcers, serological data (ANA, anti-ENA, and ET-1 concentrations), and different treatments (CA, bosentan).

Patient	DUs	Therapy	Anticentromere		Anti-ENA		ET-1 concentration (pg/mL)	
			1° serum	2° serum	1° serum	2° serum	1° serum	2° serum
(1) DfSSc, PAH	Yes	Bosentan	Neg.	Neg.	Scl 70 (127,2)	Scl 70 (130,8)	8,1	6,9
(2) DfSSc	No	CA	Neg.	Neg.	Scl 70 (46,9); SS-A (143)	Scl 70 (75,0); SS-A (143,8)	7,3	3,7
(3) LcSSc,	Yes	CA	Neg.	Neg.	Neg.	Neg.	4,6	4,6
(4) LcSSc, PAH	No	Bosentan	Pos.	Pos.	Neg.	Neg.	5,7	4,6
(5) LcSSc, PAH	Yes	Bosentan	Pos.	Pos.	Neg.	Neg.	4,8	3,7
(6) LcSSc	No	CA	Neg.	Neg.	Scl 70 (108,8)	Scl 70 (136,6)	4,6	4,4
(7) LcSSc, PAH	No	Bosentan	Pos.	Pos.	Neg.	Neg.	3,8	3,5
(8) LcSSc	Yes	CA	Neg.	Neg.	SS-A (132,1)	SS-A (133,5)	24	4,1
(9) LcSSc, PAH	No	Bosentan	Pos.	Pos.	Neg.	Neg.	6,0	4,9
(10) LcSSc	Yes	CA	Pos.	Pos.	Neg.	Neg.	4,35	4,6
(11) LcSSc	No	CA	Pos.	Pos.	SS-A (33,0)	SS-A (43,8)	5,0	4,6
(12) LcSSc	Yes	CA	Pos.	Pos.	Neg.	Neg.	4,2	4,2
(13) LcSSc	Yes	CA	Pos.	Pos.	Neg.	Neg.	4,0	3,6
(14) LcSSc	No	CA	Neg.	Neg.	Neg.	Neg.	4,0	3,7
(15) LcSSc	No	CA	Pos.	Pos.	Neg.	Neg.	4,3	3,75
(16) LcSSc	No	CA	Neg.	Neg.	Scl 70 (133,4); SS-A (41,4)	Scl 70 (134,0); SS-A (44,6)	3,3	4,5
(17) CREST	No	CA	Pos.	Pos.	Neg.	Neg.	4,3	3,2
(18) DfSSc	No	CA	Neg.	Neg.	Scl 70 (102,3); SS-A (128,1); SS-B (127,1)	Scl 70 (121,0); SS-A (133,8); SS-B (131,0)	6,4	7,2

DfSSc: diffuse scleroderma; LcSSc: limited scleroderma; PAH: pulmonary arterial hypertension; DUs: digital ulcers; ANA: anti-nuclear antibodies; anticentromere: anticentromere antibodies; anti-ENA (Scl 70, SS-A, SS-B): anti-extractable nuclear antigen antibodies; ET-1: endothelin-1; Neg.: negative; Pos.: positive.
